# Comprehensive analysis of the expression and prognosis for APOE in malignancies: A pan-cancer analysis

**DOI:** 10.32604/or.2022.026141

**Published:** 2022-12-06

**Authors:** SHOUKAI YU, LINGMEI QIAN, JUN MA

**Affiliations:** 1 Hongqiao International Institute of Medicine & Clinical Research Center, Tongren Hospital, Shanghai Jiao Tong University School of Medicine, Shanghai, China; 2 Clinical Research Institute, Shanghai Jiao Tong University School of Medicine, Shanghai, China; 3 Harvard T.H. Chan School of Public Health, Harvard University, Boston, USA

**Keywords:** APOE, Pan-cancer, Bioinformatics, Alzheimer’s disease

## Abstract

Apolipoprotein E (*APOE*), a gene identified as one of the strongest genetic factors contributing to the risk determinant of developing late-onset Alzheimer’s disease (AD), may also contribute to the risk of cancer. However, no pan-cancer analysis has been conducted specifically for the *APOE* gene. In this study, we investigated the oncogenic role of the *APOE* gene across cancers by GEO (Gene Expression Omnibus) and TCGA (The Cancer Genome Atlas). Based on the available data, we found that most cancer types overexpress *APOE*, and clear associations exist between the expression level of *APOE* and prognosis in tumor patients. The expression of *APOE* also correlates with certain gender-associated tumors including, ovarian cancer, uterine carcinosarcoma, and breast cancer. However, there is a significant negative association between cancer-associated fibroblast infiltration levels and the expression level of *APOE* in testicular germ cell tumors. Moreover, acute inflammatory response and protein-activation cascade-associated functions play an important role in the functional mechanisms of APOE. The present pan-cancer analysis of APOE shows that the protein phosphorylation, DNA methylation, and genetic alterations of APOE have a significant clinical relevance for survival prognosis and immune cell infiltration. This novel pan-cancer study outlines the current understanding of APOE oncogenic roles across thirty-three cancers and highlights the complex association between AD and cancers.

## Introduction

Cancer is one of the leading causes of death, leading to millions of deaths globally each year, despite significant advances in cancer biology and therapy. Some primary challenges in drug development include the complexity of biological systems and the lack of data. There has been a great effort to share cancer data openly to understand these diseases. For example, the Cancer Genome Atlas (TCGA) and International Cancer Genome Consortium (ICGC) have profiled molecular landscapes of 33 cancer types in over 11,100 patients. Millions of somatic mutations have been identified across human cancers, with thousands of alleles implicated in disease causation. Alzheimer’s disease (AD) is an acquired and progressive cognitive impairment and a degenerative brain disease. AD patients are estimated to be about 75 million by 2030 and 1.315 billion by 2050 [1], with anticipated continued acceleration as the aging population continues to rise. The causes of both AD and cancer are complex; importantly, these diseases are thought to be impacted by both social and biological effects, including alterations of lipoproteins.

Apolipoprotein E (APOE) is a lipoprotein encoded by the *APOE* gene and a primary lipid carrier in the brain. APOE has three common subtypes: *APOE*2, *APOE*3, and *APOE*4, encoded by the *ε2*, *ε3*, and *ε4* alleles. A primary component of amyloid plaques, APOE is the strongest risk locus for late-onset AD and promotes the deposition and accumulation of amyloid plaques. Amyloid β (Aβ) is associated with AD and can induce cytotoxicity in tumor cells [2]. Moreover, anti-APOE immunotherapy inhibits the accumulation and deposition of amyloid, which further supports the role of APOE in aβ aggregation and clearance.

APOE plays a unique role in AD, and the inverse association between AD and cancer has been previously reported [3–5]. Longitudinal [6–9] and cross-sectional [10–12] studies alike have reported statistically significant effects of the APOE genotype on AD signature cortical thickness and hippocampal volume in patients with AD, although with contradictory results [13]. Interestingly, incidence rates of AD are greater in women, and some cancers are gender-specific, including invasive breast carcinoma (BRCA), uterine *corpus* endometrial carcinoma (UCEC), and testicular germ cell tumors (TGCT). This observation indicates that both AD and cancer have a gender effect. Previous research also indicates that the inverse relationship between AD and cancer could be associated with estrogen [3–5]. However, studies investigating APOE in cancer are limited. Here, we compared APOE expression in tumors and its corresponding control tissue across about 33 different cancers.

The present study provides the first pan-cancer analysis of APOE across different cancers using datasets of the NCBI Gene Expression Omnibus (GEO) and TCGA. To further investigate the potential molecular mechanism of APOE involved in the pathogenesis of different cancer types, we analyzed gene alteration and mutation, protein phosphorylation, survival prognosis, GO enrichment, KEGG pathways, and immune infiltration. The present study carried out a thorough and necessary investigation of the specific effects of APOE on human cancers.

## Methods

### Gene expression analysis of APOE

The “Gene_DE” module of the TIMER2.0 web server (http://timer.comp-genomics.org/). TIMER2.0 represents the tumor immune estimation resource v2, and it was used to explore the differences between *APOE* mRNA levels in multiple tumor tissues and matched normal tissues by the TCGA database. There are no data available about APOE isoforms in the databases; therefore, we analyzed *APOE* expression as a whole. For tumors without normal tissue counterparts, the Genotype-tissue expression database (GTEx) was analyzed to generate plots that show the expression difference for mRNA using the GEPIA2 (Gene Expression Profiling Interactive Analysis 2, http://gepia2.cancer-pku.cn/) [14]. The cutoff values were set as a *p*-value less than 0.001, and the log2 fold change (FC) equals 2.5.

To determine the protein expression difference in *APOE* between tumor tissues and matched normal tissues, this study analyzed protein expression using the UALCAN. Ten tumor types (ovarian cancer, colon cancer, breast cancer, head, and neck squamous cell carcinoma (HNSC), endometrial cancer, renal cell cancer, pancreatic adenocarcinoma (PAAD), glioblastoma multiforme (GBM), hepatocellular carcinoma, and lung adenocarcinomas) were available for analysis. The datasets used were from The National Cancer Institute’s Clinical Proteomic Tumor Analysis Consortium (CPTAC).

The expression level of *APOE* at various pathological stages for different tumor types was examined by the “Stage-plot” panel using GEPIA2. A threshold of 0.5 was set to split the cohorts into high- and low-expression groups.

### DNA methylation analyses

The *APOE* DNA methylation was analyzed through the methylation module by TCGA and UALCAN [15,16]. There are 33 tumors available for the present analyses. Four probes of the *APOE* promoter were applied to uncover the DNA methylation levels of *APOE*: cg04406254, cg14123992, cg26190885, and cg01032398.

### Survival prognosis of APOE

The “Survival Map” panel was applied to provide figures for overall survival (OS) and disease-free survival (DFS) through GEPIA2. A threshold value of 0.5 was set to divide the high and low-expression cohorts. We utilized the log-rank test to conduct the hypothesis testing.

### Genetic alteration analysis of APOE

The cBioPortal webserver (cBio Cancer Genomics Portal, https://www.cbioportal.org/) [17,18] was applied to analyze the genetic alteration, variation, and mutation of *APOE* through the “quick search” tab and “TCGA Pan-Cancer Atlas Studies” panel. Then, this step leads to the panel of Cancer Types Summary, which demonstrates copy number alteration (CNA), together with alteration frequency and mutation type across all analyzed tumors. In addition, the “mutations” panel demonstrates the information on the mutated site for *APOE*, and the “comparison” panel demonstrates the results of the OS, DFS, and progression-free survival of TCGA cases for both altered and unaltered groups. Finally, Kaplan-Meier curves were generated with log-rank *p*-values.

### Immune infiltration analysis of APOE

The potential association between the expression level of *APOE* and immune infiltration was examined and estimated by the “Immune Estimation” function of the TIMER2. In the “Gene” module, we selected “APOE” under Gene Expression, T cell CD8+, and cancer-associated fibroblasts under Immune Infiltrates. The algorithms include TIMER, CIBERSORT-ABS, EPIC, QUANTISEQ, CIBERSORT, MCP-COUNTER, and XCELL. These methods were adapted to obtain the estimation of immune infiltration of T cell CD8+. The algorithms of EPIC, MCP-COUNTER, XCELL, and TIDE were used to obtain the estimation of immune infiltration of cancer-associated fibroblasts (CAFs).

### Gene-related enrichment analysis of APOE

In this study, we used the STRING webserver to search protein name “APOE” with the organism selection of “*Homo sapiens*” [19,20]. The STRING website was https://string-db.org/. The used parameter settings included “evidence,” “Experiment,” “low confidence (0.150)” for minimum interaction, and “no more than 50 interactors” for the max potential number of interactors to display in 1st shell. The results provide 50 proteins binding to APOE.

We used “Similar Gene Detection” to get 100 top APOE-correlated targeting genes using the TCGA dataset from GEPIA2. The “Correlation Analysis” was utilized to calculate the Pearson correlation coefficient for the selected top genes. Moreover, the log2 TPM was used for generating dot plots. Additionally, the correlation coefficients with corresponding *p*-values were also provided from GEPIA2. The “Gene_Corr” panel of TIMER2 was utilized to produce heatmaps with the *p*-value and corresponding partial correlation in Spearman’s test. Furthermore, an analysis of the APOE-binding and interacting genes were represented using Venn diagrams. Likewise, the statistical analyses and enriched pathways were analyzed with the R packages, including “clusterProfiler,” “ggplot2”, together with “tidyr”.

## Results

### APOE is overexpressed in seven tumors

**Figure 1:**
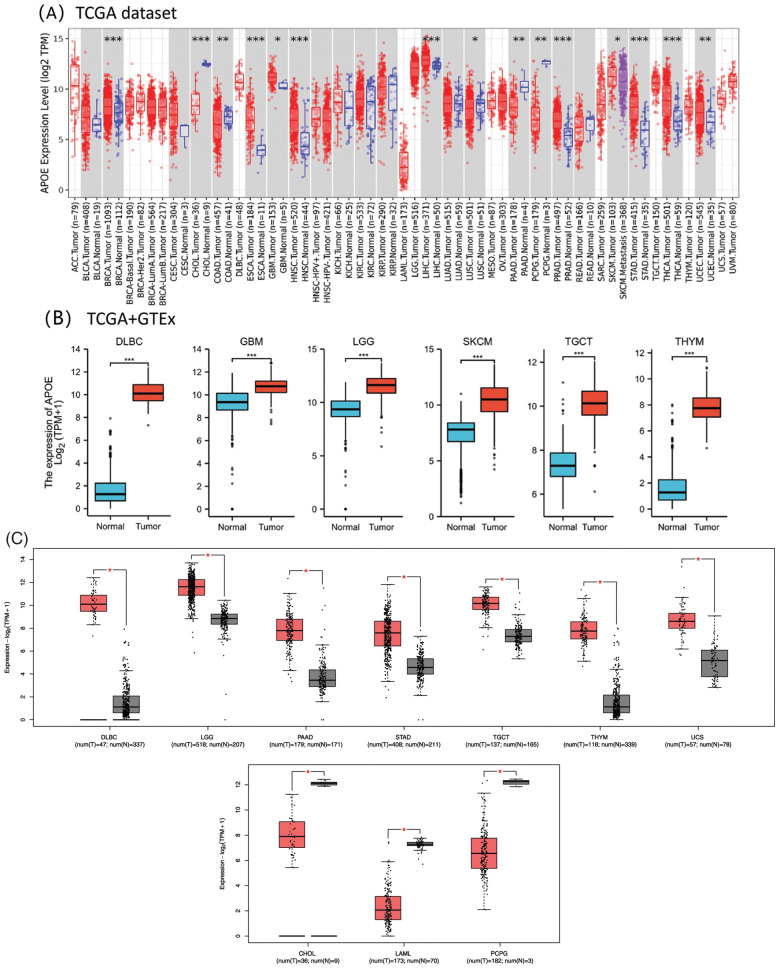
The mRNA expression profiles of *APOE* in different studies unifying data from The Cancer Genome Atlas (TCGA) and the Genome Tissue Expression Consortium Project (GTEx). (A) The expression level of *APOE* in different cancers was analyzed using TIMER2.0. with the *p*-value cut-offs of *p < 0.05; **p < 0.01; and ***p < 0.001. (B) For the six selected cancer types in the TCGA project, their matched normal tissues from the GTEx database were analyzed as normal controls. (C) The *APOE* expression profiling in different types of tumors was analyzed through GEPIA2. Red represents tumor groups, and blue represents their corresponding normal controls.

In the TCGA database, the *APOE* expression was statistically significantly higher in some tumor tissues compared to the matched control tissues, which include BRCA, HNSC, Liver hepatocellular carcinoma (LIHC), esophageal carcinoma (ESCA), prostate adenocarcinoma (PRAD), stomach adenocarcinoma (STAD), and thyroid carcinoma (THCA) (Fig. 1A, *p*-value less than 0.001). The *APOE* expression level was statistically significantly lower in certain tumor tissues than in the matched control tissues, particularly in cholangiocarcinoma (CHOL), with a *p*-value less than 0.001), and Colon adenocarcinoma (COAD), PAAD, Ovarian serous cystadenocarcinoma (OVs), and Pheochromocytoma and Paraganglioma (PCPG), all with *p* < 0.01.

Furthermore, significant expression differences of *APOE* were observed between tumor and normal tissues of GBM, Brain lower grade glioma (LGG), Lymphoid neoplasm diffuse large B-cell lymphoma (DLBC), TGCT, Skin cutaneous melanoma (SKCM), and Thymoma (THYM) (Fig. 1B, *p* < 0.001). Based on GEO datasets, APOE expression was upregulated in seven tumors (DLBC, LGG, PAAD, STAD, TGCT, THYM, and Uterine Carcinosarcoma (UCS); Fig. 1C) and downregulated in three tumors (CHOL, LAML, PCPG; Fig. 1C).

**FIGURE S1:**
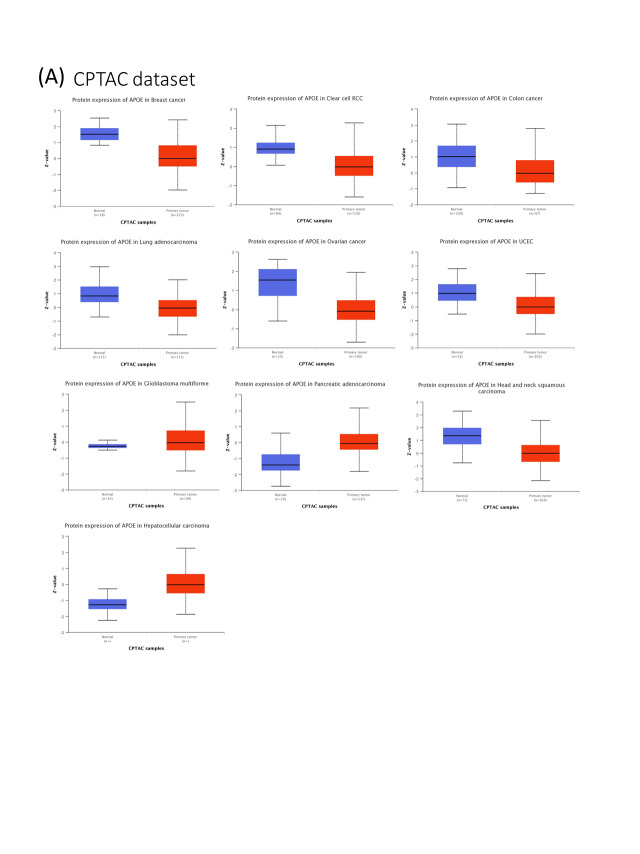
The expression of APOE in different types of tumors. (A) The expression level of APOE total protein between normal tissues and primary tumor tissues within the CPTAC program.

**FIGURE S2:**
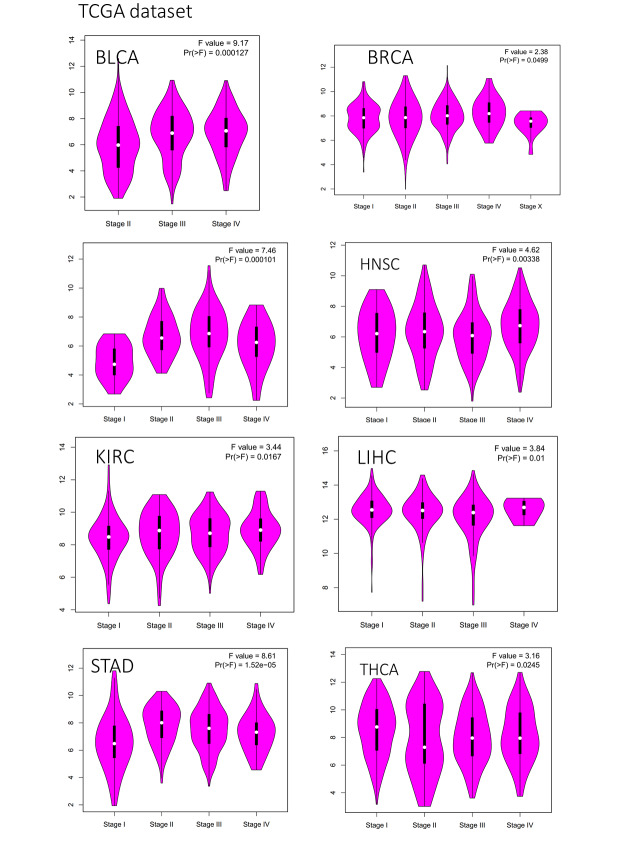
The expression levels of APOE and the different pathological stages of different tumor types. The display results of *p* < 0.05.

APOE total protein is significantly heightened in the primary tissues of some cancers, including breast cancer, colon cancer, ovarian cancer, UCEC, and LUAD (as shown in Suppl. Fig. S1, *p* < 0.001) compared to their matched normal tissues, as indicated by the CPTAC dataset analyses. The protein expression of APOE in the tumor group was decreased for breast cancer, colon cancer, renal cell cancer, ovarian cancer, endometrial cancer, HNSC, and lung adenocarcinoma, compared to their normal tissues. The results demonstrated that in the tumor group, the protein expression of APOE was increased for pancreatic adenocarcinoma and hepatocellular carcinoma compared to the corresponding normal tissues. Moreover, the expression level of APOE was found to be statistically significantly associated with the stages of cancer in BLCA, BRCA, ESCA, HNSC, Kidney renal clear cell carcinoma (KIRC), LIHC, STAD, and THCA (Suppl. Fig. S2, all *p*-values are less than 0.05), as determined using GEPIA2. The later stages of tumor progression tend to have higher expression levels of APOE, except for THCA. A decrease in expression was observed for most of the analyzed tumors, except for PAAD and Hepatocellular carcinoma.

### Overexpression of APOE predicts a better prognosis in gliomas

**Figure 2:**
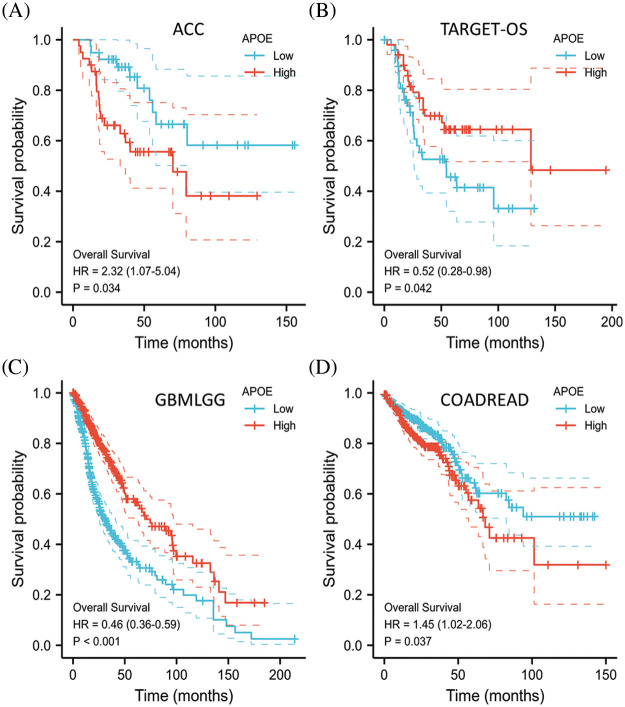
Overall survival (OS) of malignant tumors with APOE abnormal expression. Higher APOE expression represented a better survival prognosis in the GBMLGG and TARGET-OS data, while higher APOE expression in ACC and COADREAD indicated a worse prognosis.

We next investigated the effect of APOE expression on OS and DFS. To date, no statistical association has been conducted between the expression levels of APOE and the patient prognosis in DFS. Higher APOE expression indicated better prognosis in the GBMLGG and TARGET-OS groups after OS analysis (Fig. 2). However, higher APOE expression in ACC and COADREAD show unfavorable results, indicating that APOE may have both positive and negative effects on a patient’s survival outcomes.

### Amplifications are the most frequent genetic alterations of APOE

**Figure 3:**
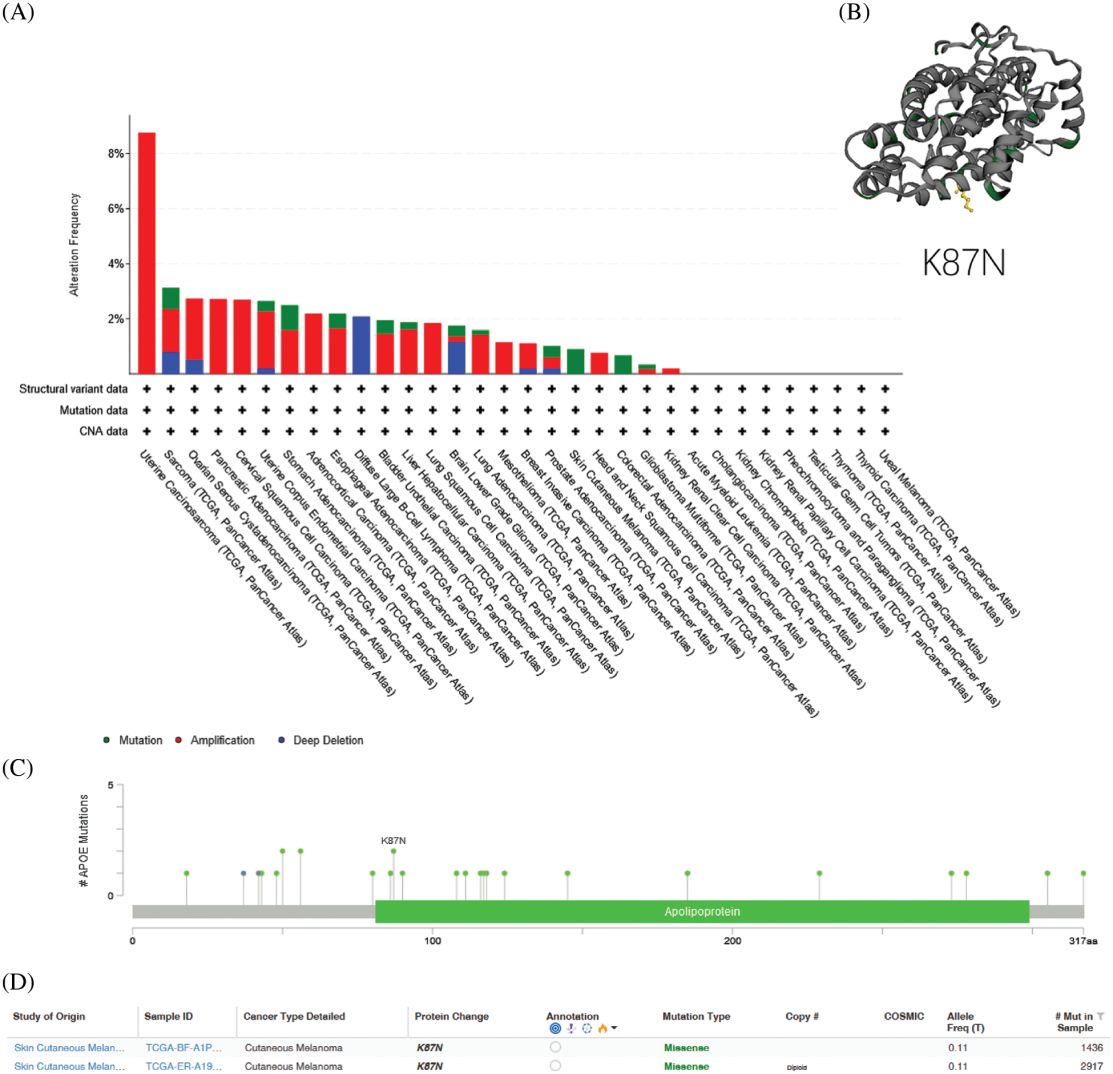
Genetic variation of *APOE* in different types of tumors. (A) Genetic variation of *APOE* in different types of tumors through the cBioPortal site. There are 32 studies with a total of 10,953 patients with 10,967 samples. The altered/profiled ratio is 149/10950. (B) The most observed mutation was K87N in the TCGA cohort, which was predicted to be inactivating mutation.

Amplifications of *APOE*, the most frequent DNA alteration in the TCGA pan-cancer panel, were observed primarily in UCEC, OV, and BRCA patients (Fig. 3). In contrast, *APOE* deletions in malignancies were sparsely distributed along the functional region of *APOE* without apparent hot spot sites of mutation (Fig. 3). The K87N was the most frequent mutation (Fig. 3), a potential inactivating mutation.

Genetic alterations of *APOE* were investigated across various cancer samples in the cohorts from TCGA. The analyses of *APOE* demonstrated that the most common DNA alteration was the observed “amplification” of CNA from TCGA pan-cancer, as demonstrated using the cBioPortal website. Amplifications were primarily observed in UCS, Cervical Squamous Cell Carcinoma (CESC), Adrenocortical Carcinoma (ACC), Lung Squamous Cell Carcinoma (LUSC), Mesothelioma (MESO), HNSC, PAAD, and KIRC (Fig. 3).

The overall mutation rate of *APOE* was relatively low (less than 1%) across all cancer types. The highest frequency of *APOE* mutation changes (0.91%) was observed in STAD patients, followed closely by BLCA (0.49%) (Fig. 3). UCS patients tend to have high levels of CAN amplification, with a frequency change of 8.77%. No hot spot mutation sites for *APOE* were observed in the current dataset. However, the most frequent alteration in the Apolipoprotein region was demonstrated from the three-dimensional model of APOE (Fig. 3).

**FIGURE S3:**
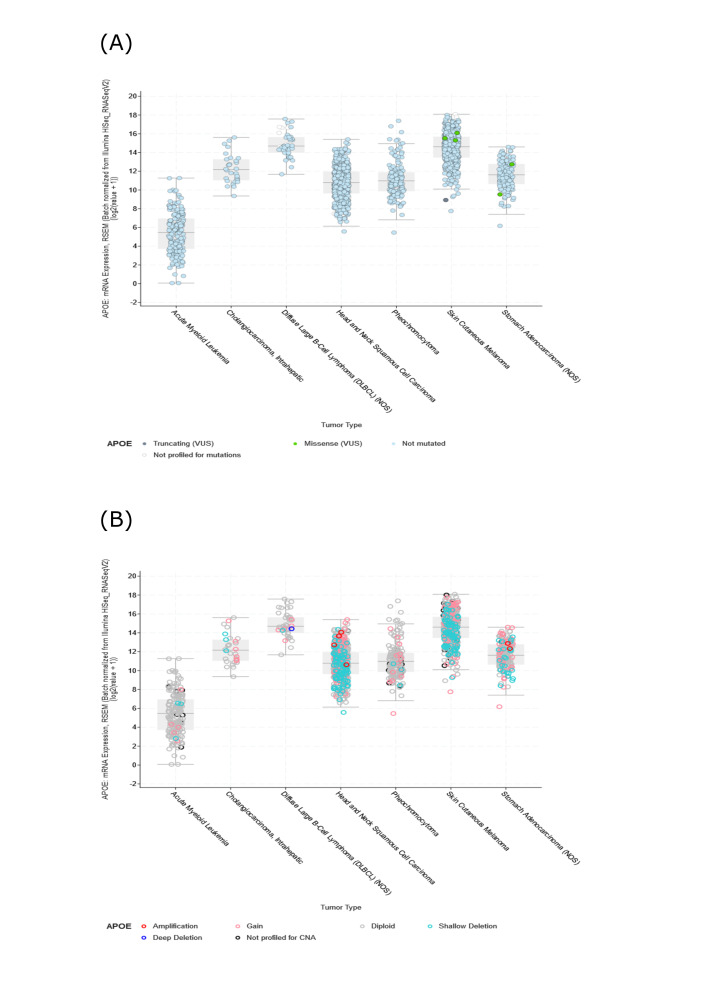
The relationship between the expression level of APOE and genetic disorders. (A) The mutations of the APOE gene were not directly relevant to the RNA expression of APOE. (B) The copy variations were not directly related to the APOE RNA expression.

We also examined the association between genetic variation and the expression of *APOE*. The cBioportal analyses indicated that the observed mutations were not directly associated with *APOE* RNA expression (Suppl. Fig. S3A). Similarly, there was no direct relationship between DNA copy variations and APOE expression (Suppl. Fig. S3B). This result indicates that the expression of *APOE* was not due to genetic alterations.

Finally, the potential association between genetic variations of *APOE* and the patient prognosis was also investigated. Results indicate that the presence of genetic alterations of *APOE* cannot be used to predict the patient’s prognosis.

### DNA methylation of APOE varies across different cancers

**FIGURE S4:**
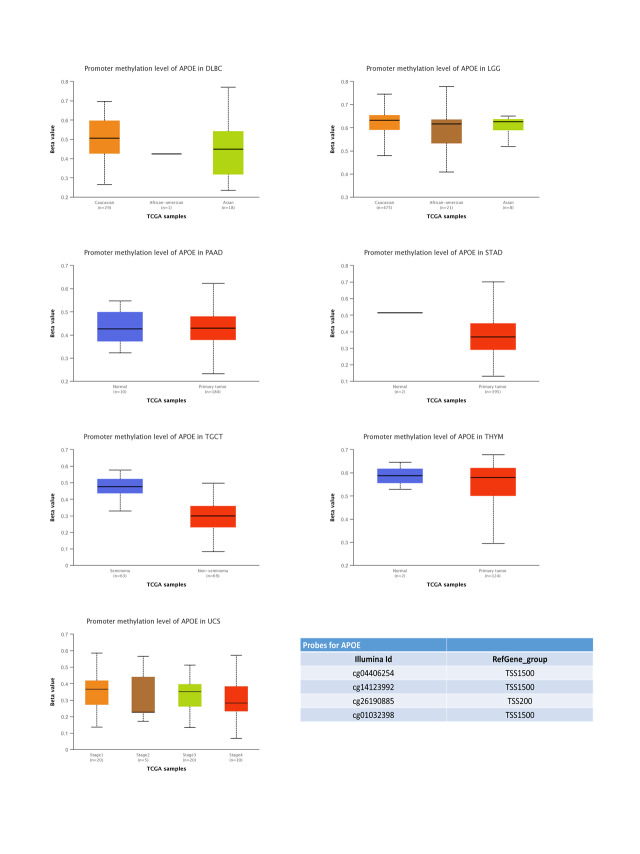
The upregulated APOE DNA methylation level in different types of tumors. Four probes were used to detect the DNA methylation level of the *APOE* promoter. The data were obtained using UALCAN.

**FIGURE S5:**
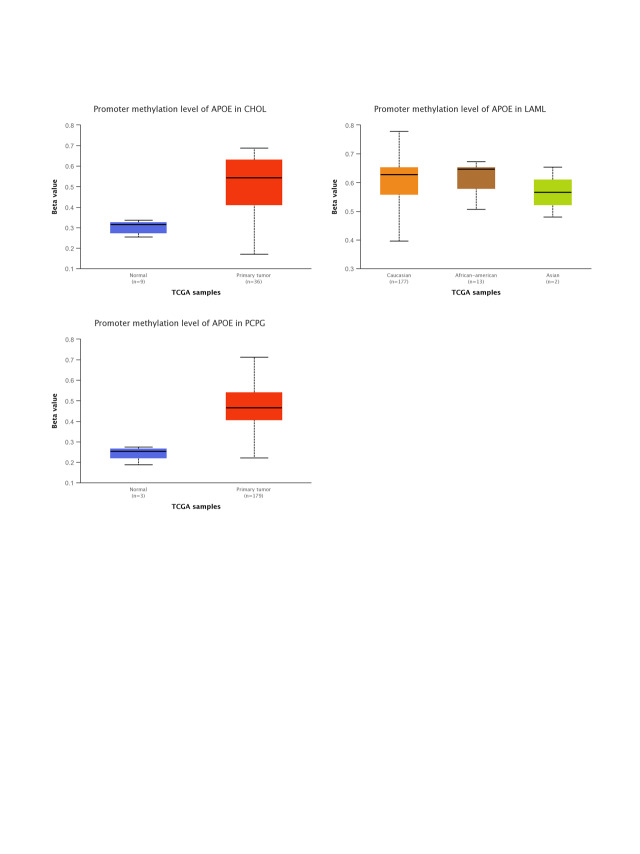
The downregulated DNA methylation level of APOE in different types of tumors. Four probes were used to detect the DNA methylation level of the *APOE* promoter. The data were obtained using UALCAN.

In this study, we used four probes in the *APOE* promoter to detect the potential methylation levels of APOE (Suppl. Figs. S4 and S5). The DNA methylation level of *APOE* did not change for the seven upregulated and three downregulated tumors. Because no available DNA methylation dataset exists for normal controls for tumors DLBC, LGG, SKCM, UCS, and LAML, no direct comparison was conducted for the global DNA methylation level. Instead, this study compared DNA methylation levels across different populations (Caucasian, African-American, and Asian) or tumor stages (Stage 1 to Stage 4). However, no change in levels of DNA methylation was observed. Together, these results indicate that DNA methylation does not play a key part in abnormal APOE expression.

### Phosphorylation levels of APOE vary across different cancers

**FIGURE S6:**
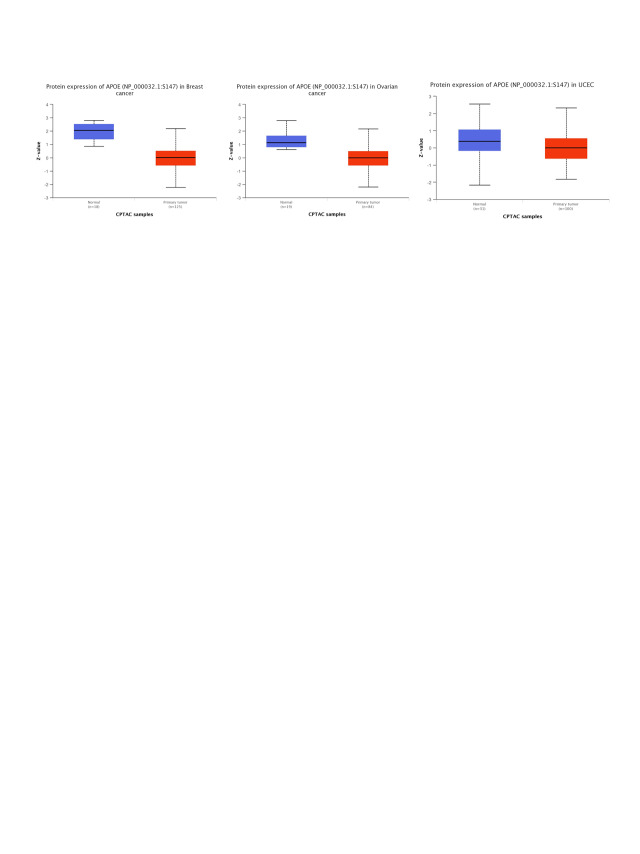
The results of phosphorylation analysis for APOE in different types of tumors through the CPTAC program and the UALCAN.

We compared the phosphorylation levels of APOE in primary tumor tissue and their matched normal tissue by CPTAC for three types of tumors: breast cancer, ovarian cancer, and UCEC. The phosphorylation site at the S147 locus of APOE significantly differs from the control group for all three examined cancer types (Suppl. Fig. S6). Notably, the S147 locus has a statistical significance in the declined phosphorylation level of primary tumor tissues compared to their matched normal tissues in breast cancer with the *p*-value of 4.61e-13 and ovarian cancer with the *p*-value of 1.07e-06, but not for UCEC (*p* = 1.65e-01).

### A positive correlation was identified between immune infiltration analyses and APOE

**Figure 4:**
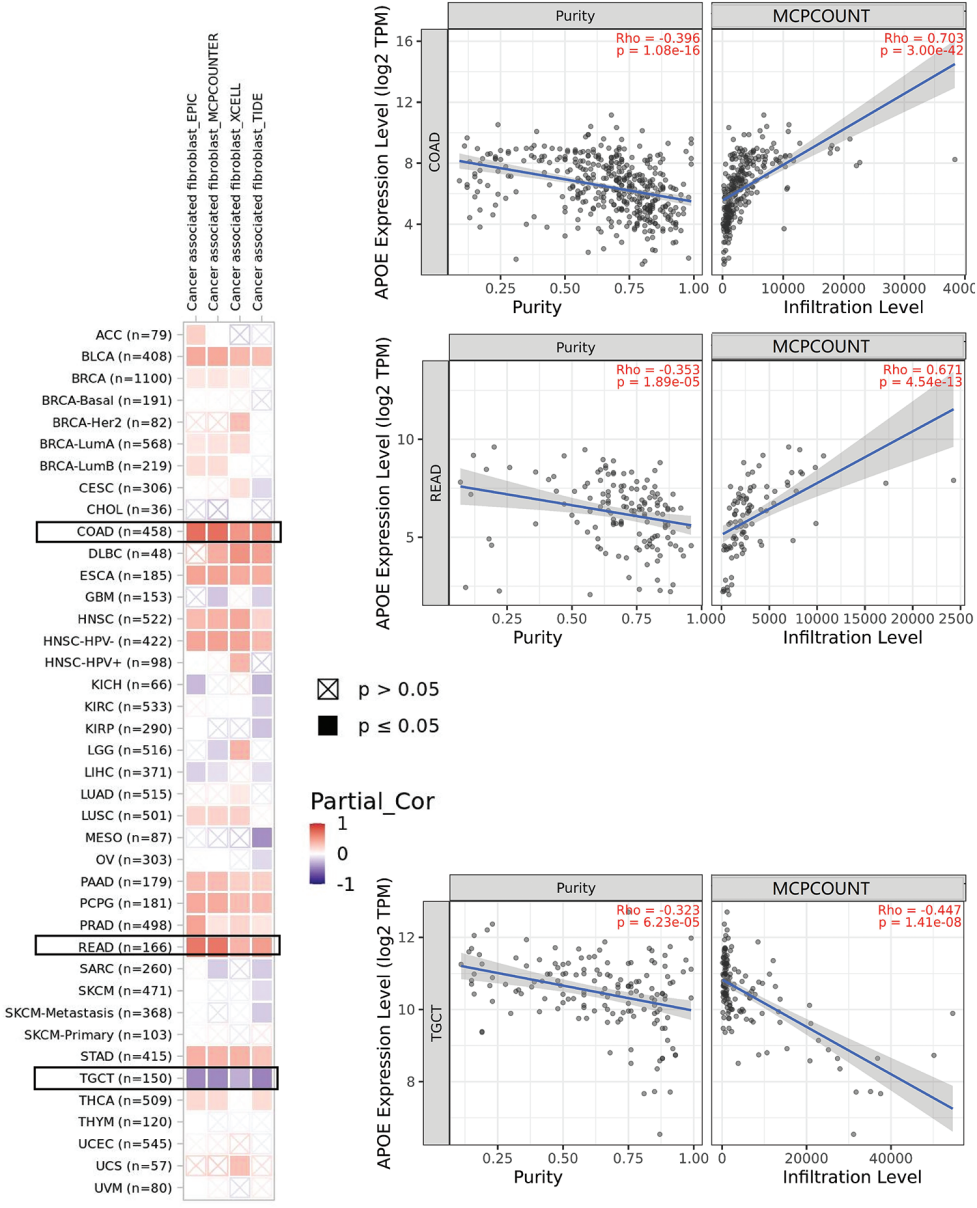
The potential association among APOE expression and immune infiltration of cancer-associated fibroblasts (CAFs). Four algorithms were utilized to explore the associations between APOE expressions and the infiltration of CAFs in different cancers. The correlation and scatterplot for three selected cancer types are shown in the right panels.

**FIGURE S7:**
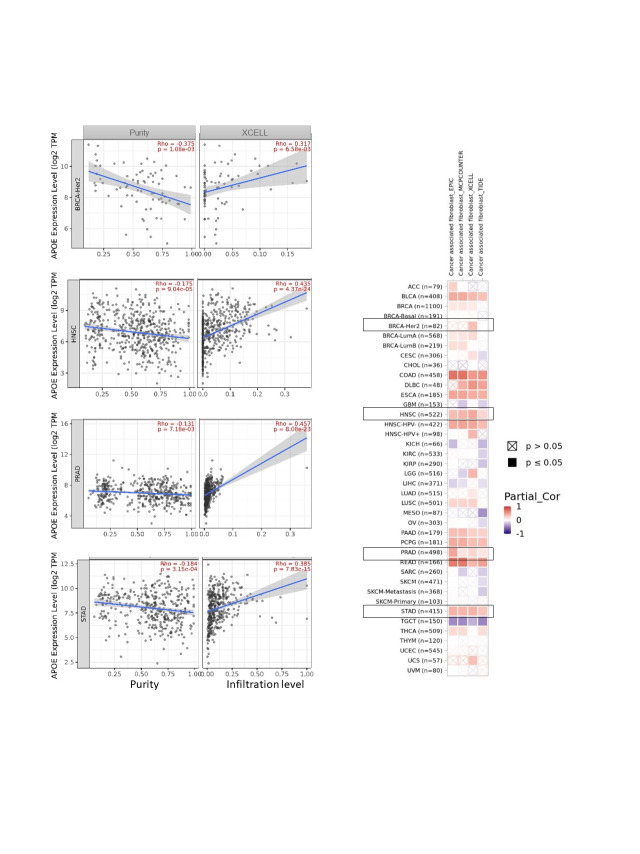
The association among the expression levels of APOE and immune infiltration of cancer-associated fibroblasts (CAFs). Four algorithms were utilized to explore the association between the expression levels of APOE and the infiltration of CAFs in different types of cancers.

**FIGURE S8:**
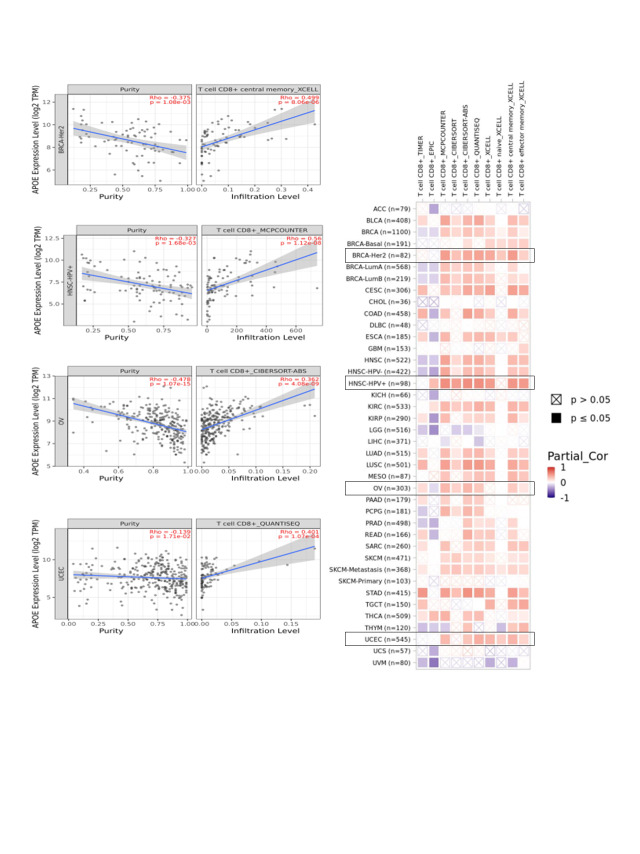
The association among the expression levels of APOE and immune infiltration of T cell CD8+. Ten algorithms were utilized to explore the association between APOE expression and infiltration of T cell CD8+ in different types of cancers.

The algorithms of TIMER, EPIC, MCP-COUNTER, CIBERSORT, CIBERSORT-ABS, QUANTISEQ, XCELL, and TIDE were applied to explore the correlations among the infiltration level of immune cells, which include T cell CD8+ and CAFs using TCGA and APOE expression for different cancer types (Suppl. Figs. S7 and S8). APOE expression in COAD, for example, is statistically significantly positively associated with the infiltration level of CAFs (R = 0.703, *p* = 3e-42, Fig. 4) according to the MCPCOUNTER algorithm. Significant positive correlations were also observed among the expression level of APOE and the immune infiltration of CAFs in BLCA, COAD, HNSC, PAAD, PCPG, ESCA, READ, and STAD by TCGA.

### Enrichment pathways identified for APOE

**Figure 5:**
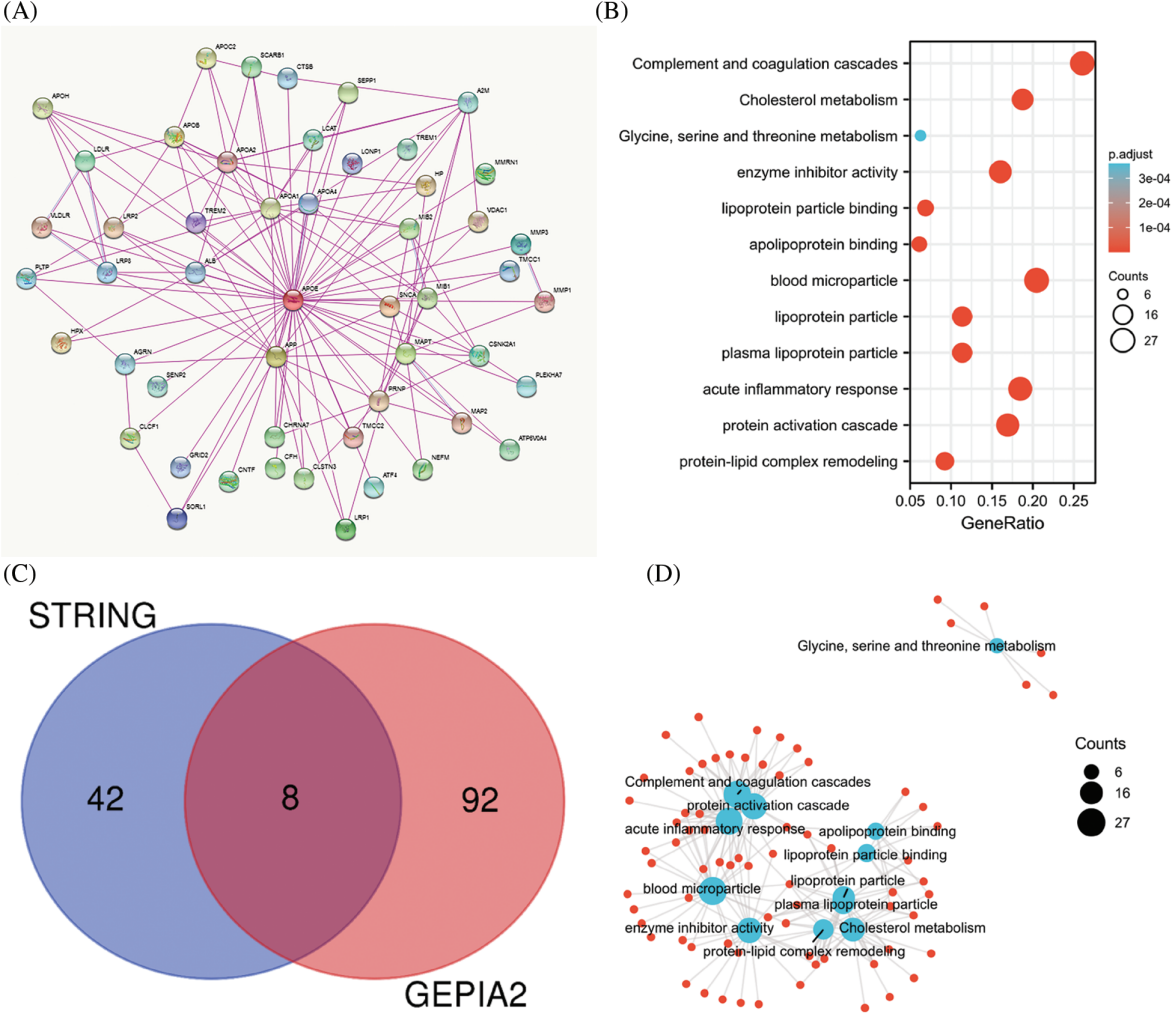
Results of pathway enrichment analysis of APOE. (A) The STRING analysis identified a total of 50 proteins that bind to APOE. There were 100 genes in the TCGA database associated with APOE. (B) The results from the KEGG pathway analysis were calculated from the APOE-binding and interacting genes. (C) The intersection graph of the APOE-binding and APOE-correlated genes. (D) The cnetplot for gene oncology analysis.

**FIGURE S9:**
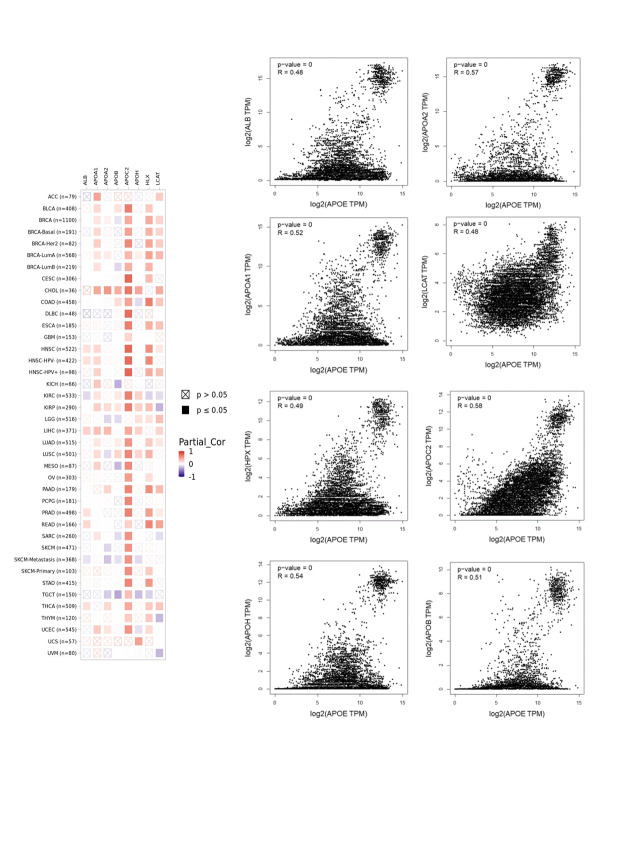
(A) Heatmap results for selected types of cancers. (B) The correlations among the expression levels of APOE and selected targeted genes.

To investigate the potential molecular mechanism of APOE in tumorigenesis, the present study screened the genes related to the expression of APOE and the targeting of APOE-binding protein for pathway enrichment. Fifty APOE-binding proteins were analyzed by the STRING analysis, forming an interaction network. The 100 top genes related to the expression of APOE were calculated using GEPIA2 based on all tumor data by TCGA. These above datasets share eight common members (ALB, APOA1, APOA2, APOB, APOC2, APOH, HPX, and LCAT), some of which are positively correlated across cancer types (Suppl. Fig. S9). There is a positive correlation between APOE and APOA1 across many of the analyzed cancers (Fig. 5).

The two data sets were combined to conduct further KEGG and GO enrichment analysis. This study demonstrates that enrichment accentuates “acute inflammatory response,” “protein-activation cascade” in biological processes GO components (BP), “blood microparticle” in cellular components GO components (CC), and “Complement and coagulation cascades” in KEGG pathway database (Fig. 5).

## Discussion

*APOE* gene polymorphism is the main genetic risk determinant of late-onset AD (20), and different alleles of *APOE* play different roles in AD development [21–23] and alter in frequency in the general population. The most common allele (*e3*) is found in more than half of the general population, and the next most common, *e4*, increases the risk of developing AD. Although AD and cancer are associated with age, previous studies [3–5,24–26] have demonstrated an inverse relationship between cancer and AD in several aspects, including estrogen, P53 expression, neurotrophins and growth factors (NGF), epidermal growth factor receptor (EGFR), cAMP, and PI3K/AKT/mTOR pathway, among others. Based on the available data, this study analyzed APOE expression.

The phosphorylation analyses using the CPTAC dataset were available for three cancer types: breast cancer, ovarian cancer, and UCEC. Phosphorylation levels of S147 locus tumors decreased for all three cancer types. Still, they were only statistically significant for breast cancer (*p* = 4.61e-13) and ovarian cancer (*p* = 1.07e-06) compared to the normal corresponding control group. The APOE protein level in the primary tumor was lower than in corresponding normal tissue for seven cancer types and higher for three cancer types (Glioblastoma multiforme, PAAD, and Hepatocellular carcinoma) in the UALCAN databases (Suppl. Fig. S1, all *p* < 0.01). This posttranslational modification site’s clinical and biological significance remains to be determined. One possibility is that the statistical significance in decreased levels of APOE phosphorylation at the S147 locus might serve as signals of dysregulated functional patterns in tumor samples. Additional research is required to fully understand the specific roles of S147 phosphorylation of APOE and cell cycle regulation in tumorigenesis.

In this study, the mRNA expression levels of APOE were significantly increased for seven out of the top ten cancers containing genetic alterations (Figs. 1 and 3), most of which were amplifications. This result indicates the potential positive correlation between mRNA expression levels and genetic amplifications. Furthermore, the non-significant results for mutation and DNA copy number variants (Suppl. Fig. S3) also confirm the lack of direct correlation between mRNA levels of *APOE* and genetic alterations. However, it is worth noting that APOE has been previously identified as a regulator in tumorigenesis [27,28]. The DFS and OS analyses also indicate the different roles played by APOE across cancer types. However, for many tumors the prognosis is difficult to obtain from the expression level of APOE alone. In addition, previous research indicated that diet could impact the levels of APOE expression [29,30].

Although the protein expression levels of APOE cannot be predicted from the mRNA level of APOE directly, the decreased protein expression levels of APOE were regulated in female-dominant cancer types, such as breast cancer, ovarian cancer, and UCEC (as shown in Suppl. Fig. S1). Relatedly, the incidence of AD in females is twice that of males, further suggesting a potential inverse relationship between AD and some cancer types. Furthermore, lower protein expression levels of APOE were obtained for clear cell renal carcinoma, colon cancer, LUAD, and HNSC, an observation confirmed by phosphorylation analyses (Suppl. Fig. S6). Finally, we report a negative relationship between APOE expression and immune infiltration of CAFs for TGCT. However, the relationship between APOE expression and immune infiltration of CAFs for PRAD was positive. A statistically significant positive association among the expression levels of APOE and immune infiltration of T cell CD8+ for BRCA-Her2, CESC, OV, and UCEC was also observed, suggesting the high APOE expression in those tumors may impact the formation and function of some immune cells.

The immune microenvironment plays an important part in cancer initiation, progression, or elimination. A wide range of algorithms was used to explore the potential association between the immune infiltration levels of CD8+ T-cells and APOE expressions in certain tumors, such as BLCA, CESC, LUAD, LUSC, OV, STAD, THCA, and UCEC (Suppl. Fig. S8). These results are the first to reveal a positive correlation among the expression levels of APOE and the estimations of infiltration value of CAFs in some tumors. This result includes the tumors of BLCA, COAD, ESCA, HNSC, PAAD, PCPG, READ, and STAD based on the results from these algorithms (Fig. 4 and Suppl. Fig. S7).

We now address possible limitations and further directions of the current study. First, this study evaluated the mRNA level of APOE, and associated protein levels should be verified in future studies. Second, other possibilities, including histone modifications and glycosylation, may lead to abnormal expression of *APOE*, which requires further exploration. In addition, the racial, gender, and age might contribute to the expression of *APOE*. Third, results need further validation in larger cohorts because the current study was based on open-source databases. Fourth, the physiology and pathology of *ε2* and *ε4* have been reported. Their effects on *APOE* expression and blood level could be different. The effect of rare isoforms might dilute the analysis of the total *APOE* level. Fifth, systematic biases may exist in the data and respective analyses due to the retrieval of various information from different databases. Additional research is required to investigate the specific roles of APOE in different cancers and Alzheimer’s therapy.

## Conclusion

This pan-cancer analysis of APOE shows that the genetic alterations, protein phosphorylation, and DNA methylation of APOE has significant clinical relevance for survival prognosis and immune cell infiltration. Both cancers and AD are correlated with aging, but there is a potential inverse relationship for certain cancers. Based on currently available data, we analyzed *APOE* expression as a whole. This study’s results show that the total protein level of APOE and the phosphorylation level of APOE at the S147 site in the primary tumor were higher than in the normal control group. Further investigation is required to assess the roles of APOE phosphorylation at S147 and the related role of cell cycle regulation in tumorigenesis. This study demonstrates that the expression level of APOE is statistically significantly related to gene expression of immunity-related genes across different cancers.

## Supplementary Materials

## Data Availability

All the data used in this study were obtained from publicly available databases, and the data analyzed in the present study are available on request.

## List of Abbreviations

APOE: Apolipoprotein E
TCGA: The Cancer Genome Atlas
UCEC: Uterine *Corpus* Endometrial Carcinoma
TGCT: Testicular Germ Cell Tumors
GEO: Gene Expression Omnibus
GTEx: Genotype-tissue expression
AD: Alzheimer’s disease
SP: Senile plaques
GEPIA2: Gene Expression Profiling Interactive Analysis 2
CPTAC: Clinical Proteomic Tumor Analysis Consortium
FC: Fold change
CAFs: Cancer-associated fibroblasts
OS: Overall survival
DFS: Disease-free survival
CAN: Copy number alteration
BRCA: Breast invasive carcinoma
ESCA: Esophageal carcinoma
LIHC: Liver hepatocellular carcinoma
PRAD: Prostate adenocarcinoma
STAD: Stomach adenocarcinoma
HNSC: Head and neck squamous cell carcinoma
THCA: Thyroid carcinoma
CHOL: Cholangiocarcinoma
COAD: Colon adenocarcinoma
LUAD: Lung adenocarcinoma
OVs: Ovarian serous cystadenocarcinoma
PCPG: Pheochromocytoma and Paraganglioma
PAAD: Pancreatic adenocarcinoma

## References

[1] Chen, F., Chandrashekar, D. S., Varambally, S., Creighton, C. J. (2019). Pan-cancer molecular subtypes revealed by mass-spectrometry-based proteomic characterization of more than 500 human cancers. Nature Communications*,* 10 *(*1*),* 5679. DOI 10.1038/s41467-019-13528-0. PMC6908580 31831737

[2] Qin, H., Cui, T., Liu, Z., Zhou, Y., Niu, J. et al. (2021). Engineering amyloid aggregation as a new way to eliminate cancer stem cells by the disruption of iron homeostasis. Nano Letters*,* 21 *(*17*),* 7379–7387. DOI 10.1021/acs.nanolett.1c02734. 34436904

[3] Driver, J. A., Beiser, A., Au, R., Kreger, B. E., Splansky, G. L. et al. (2012). Inverse association between cancer and Alzheimer’s disease: Results from the Framingham Heart Study. BMJ*,* 344*,* e1442. DOI 10.1136/bmj.e1442. 22411920 PMC3647385

[4] Musicco, M., Adorni, F., di Santo, S., Prinelli, F., Pettenati, C. et al. (2013). Inverse occurrence of cancer and Alzheimer disease: A population-based incidence study. Neurology*,* 81 *(*4*),* 322–328. DOI 10.1212/WNL.0b013e31829c5ec1. 23843468

[5] Shafi, O. (2016). Inverse relationship between Alzheimer’s disease and cancer, and other factors contributing to Alzheimer’s disease: A systematic review. BMC Neurology*,* 16 *(*1*),* 236. DOI 10.1186/s12883-016-0765-2. 27875990 PMC5120447

[6] Mori, E., Lee, K., Yasuda, M., Hashimoto, M., Kazui, H. et al. (2002). Accelerated hippocampal atrophy in Alzheimer’s disease with apolipoprotein E ε4 allele. Annals of Neurology*,* 51 *(*2*),* 209–214. DOI 10.1002/ana.10093. 11835377

[7] van de Pol, L. A., van der Flier, W. M., Korf, E. S., Fox, N. C., Barkhof, F. et al. (2007). Baseline predictors of rates of hippocampal atrophy in mild cognitive impairment. Neurology*,* 69 *(*15*),* 1491–1497. DOI 10.1212/01.wnl.0000277458.26846.96. 17923611

[8] Hostage, C. A., Choudhury, K. R., Murali Doraiswamy, P., Petrella, J. R., Alzheimer’s Disease Neuroimaging Initiative (2014). Alzheimer’s disease neuroimaging initiative, mapping the effect of the apolipoprotein E genotype on 4-year atrophy rates in an Alzheimer disease-related brain network. Radiology*,* 271 *(*1*),* 211–219. DOI 10.1148/radiol.13131041. 24475827 PMC4263636

[9] Geroldi, C., Pihlajamäki, M., Laakso, M. P., DeCarli, C., Beltramello, A. et al. (1999). APOE-ε4 is associated with less frontal and more medial temporal lobe atrophy in AD. Neurology*,* 53 *(*8*),* 1825–1832. DOI 10.1212/WNL.53.8.1825. 10563634

[10] Hashimoto, M., Yasuda, M., Tanimukai, S., Matsui, M., Hirono, N. et al. (2001). Apolipoprotein E ε4 and the pattern of regional brain atrophy in Alzheimer’s disease. Neurology*,* 57 *(*8*),* 1461–1466. DOI 10.1212/WNL.57.8.1461. 11673590

[11] Liu, Y., Paajanen, T., Westman, E., Wahlund, L. O., Simmons, A. et al. (2010). Effect of APOE ε4 allele on cortical thicknesses and volumes: The AddNeuroMed study. Journal of Alzheimer’s Disease*,* 21 *(*3*),* 947–966. DOI 10.3233/JAD-2010-100201. 20693633

[12] Mishra, S., Blazey, T. M., Holtzman, D. M., Cruchaga, C., Su, Y. et al. (2018). Longitudinal brain imaging in preclinical Alzheimer disease: Impact of APOE ε4 genotype. Brain*,* 141 *(*6*),* 1828–1839. DOI 10.1093/brain/awy103. 29672664 PMC5972633

[13] Schuff, N., Woerner, N., Boreta, L., Kornfield, T., Shaw, L. M. et al. (2009). MRI of hippocampal volume loss in early Alzheimer’s disease in relation to ApoE genotype and biomarkers. Brain*,* 132 *(*4*),* 1067–1077. DOI 10.1093/brain/awp007. 19251758 PMC2668943

[14] Tang, Z., Kang, B., Li, C., Chen, T., Zhang, Z. (2019). GEPIA2: An enhanced web server for large-scale expression profiling and interactive analysis. Nucleic Acids Research*,* 47 *(*W1*),* W556–W560. DOI 10.1093/nar/gkz430. 31114875 PMC6602440

[15] Men, C., Chai, H., Song, X., Li, Y., Du, H. et al. (2017). Identification of DNA methylation associated gene signatures in endometrial cancer via integrated analysis of DNA methylation and gene expression systematically. Journal of Gynecologic Oncology*,* 28 *(*6*),* e83. DOI 10.3802/jgo.2017.28.e83. 29027401 PMC5641533

[16] Shinawi, T., Hill, V. K., Krex, D., Schackert, G., Gentle, D. et al. (2013). DNA methylation profiles of long- and short-term glioblastoma survivors. Epigenetics*,* 8 *(*2*),* 149–156. DOI 10.4161/epi.23398. 23291739 PMC3592900

[17] Cerami, E., Gao, J., Dogrusoz, U., Gross, B. E., Sumer, S. O. et al. (2012). The cBio cancer genomics portal: An open platform for exploring multidimensional cancer genomics data. Cancer Discovery*,* 2 *(*5*),* 401–404. DOI 10.1158/2159-8290.CD-12-0095. 22588877 PMC3956037

[18] Gao, J., Aksoy, B. A., Dogrusoz, U., Dresdner, G., Gross, B. et al. (2013). Integrative analysis of complex cancer genomics and clinical profiles using the cBioPortal. Science Signaling*,* 6 *(*269*),* pl1. DOI 10.1126/scisignal.2004088. 23550210 PMC4160307

[19] Szklarczyk, D., Gable, A. L., Lyon, D., Junge, A., Wyder, S. et al. (2019). STRING v11: Protein-protein association networks with increased coverage, supporting functional discovery in genome-wide experimental datasets. Nucleic Acids Research*,* 47 *(*D1*),* D607–D613. DOI 10.1093/nar/gky1131. 30476243 PMC6323986

[20] Szklarczyk, D., Gable, A. L., Nastou, K. C., Lyon, D., Kirsch, R. et al. (2021). The STRING database in 2021: Customizable protein-protein networks, and functional characterization of user-uploaded gene/measurement sets. Nucleic Acids Research*,* 49 *(*D1*),* D605–D612. DOI 10.1093/nar/gkaa1074. 33237311 PMC7779004

[21] Boehm-Cagan, A., Bar, R., Liraz, O., Bielicki, J. K., Johansson, J. O. et al. (2016). ABCA1 agonist reverses the ApoE4-driven cognitive and brain pathologies. Journal of Alzheimer’s Disease*,* 54 *(*3*),* 1219–1233. DOI 10.3233/JAD-160467. 27567858

[22] Mahley, R. W., Weisgraber, K. H., Huang, Y. (2006). Apolipoprotein E4: A causative factor and therapeutic target in neuropathology, including Alzheimer’s disease. PNAS*,* 103 *(*15*),* 5644–5651. DOI 10.1073/pnas.0600549103. 16567625 PMC1414631

[23] Asaro, A., Carlo-Spiewok, A. S., Malik, A. R., Rothe, M., Schipke, C. G. et al. (2020). Apolipoprotein E4 disrupts the neuroprotective action of sortilin in neuronal lipid metabolism and endocannabinoid signaling. Alzheimer’s & Dementia*,* 16 *(*9*),* 1248–1258. DOI 10.1002/alz.12121. 32588544

[24] Lanni, C., Masi, M., Racchi, M., Govoni, S. (2021). Cancer and Alzheimer’s disease inverse relationship: An age-associated diverging derailment of shared pathways. Molecular Psychiatry*,* 26 *(*1*),* 280–295. DOI 10.1038/s41380-020-0760-2. 32382138

[25] Zabłocka, A., Kazana, W., Sochocka, M., Stańczykiewicz, B., Janusz, M. et al. (2021). Inverse correlation between Alzheimer’s disease and cancer: Short overview. Molecular Neurobiology*,* 58 *(*12*),* 6335–6349. DOI 10.1007/s12035-021-02544-1. 34523079 PMC8639554

[26] Roe, C. M., Fitzpatrick, A. L., Xiong, C., Sieh, W., Kuller, L. et al. (2010). Cancer linked to Alzheimer disease but not vascular dementia. Neurology*,* 74 *(*2*),* 106–112. DOI 10.1212/WNL.0b013e3181c91873. 20032288 PMC2809029

[27] Zhao, Z., Zou, S., Guan, X., Wang, M., Jiang, Z. et al. (2018). Apolipoprotein E overexpression is associated with tumor progression and poor survival in colorectal cancer. Frontiers in Genetics*,* 9*,* 650. DOI 10.3389/fgene.2018.00650. 30631342 PMC6315167

[28] Ostendorf, B. N., Bilanovic, J., Adaku, N., Tafreshian, K. N., Tavora, B. et al. (2020). Common germline variants of the human APOE gene modulate melanoma progression and survival. Nature Medicine*,* 26 *(*7*),* 1048–1053. DOI 10.1038/s41591-020-0879-3. PMC8058866 32451497

[29] Chiba, T., Kondo, Y., Shinozaki, S., Kaneko, E., Ishigami, A. et al. (2006). A selective NFκB inhibitor, DHMEQ, reduced atherosclerosis in ApoE-deficient mice. Journal of Atherosclerosis and Thrombosis*,* 13 *(*6*),* 308–313. DOI 10.5551/jat.13.308. 17192695

[30] Ma, J., Zhang, Y., Sugai, T., Kubota, T., Keino, H. et al. (2021). Inhibition of cellular and animal inflammatory disease models by NF-κB inhibitor DHMEQ. Cells*,* 10 *(*9*),* 2271. DOI 10.3390/cells10092271. 34571920 PMC8466912

